# Laryngeal mask as a safe and effective ventilatory device during Blue Dolphin tracheostomy in the ICU

**DOI:** 10.1186/cc12107

**Published:** 2013-03-19

**Authors:** A De Nicola, MJ Sucre, G Donnarumma, A Corcione

**Affiliations:** 1 San Leonardo Hospital, Castellammare di Stabia Italy; 2 Monaldi Hospital, Naples, Italy

## Introduction

Usually percutaneous tracheostomy is accomplished via the tracheal tube. Some severe complications during percutaneous dilatational tracheostomy (PDT) may be related to poor visualization of tracheal structures. The alternative implies extubation and reinsertion of a laryngeal mask (LMA). An accidental extubation as well as an injuring of the vocal cords (because of the inflated cuff during dislocation) appears impossible in this method. Subjectively, the bronchoscopic view obtained via a LMA seems to be better than that obtained with an endotracheal tube (ET) [1,2].

## Methods

In this prospective observational study, the bedside PDT was performed using the Ciaglia Blue Dolphin method in 150 critically ill patients. The patient's tracheal tube was exchanged for a LMA Fastrach™ before undertaking PDT. The insertion of the LMA, the quality of ventilation, the blood gas values, the view of the tracheal puncture site, and the view of the balloon dilatation were rated as follows: very good (1), good (2), barely acceptable (3), poor (4), and very poor (5).

## Results

PDTs with LMA were successful in 99.3% of the patients (*n = *149). The ratings were 1 or 2 in 96% of cases with regards to ventilation and to blood gas analysis, in 96.6% for identification of relevant structures and tracheal puncture site, and in 93.3% for the view inside the trachea during PDT. A rating of 5 was assigned to one patient requiring tracheal reintubation for inadequate ventilation. There were no damages to the bronchoscope or reports of gastric aspiration.

## Conclusion

The Blue Dolphin PDT using a LMA showed definite advantages regarding inspection of dilation process. This method improves visualization of the trachea and larynx during a video-assisted procedure and prevents the difficulties associated with the use of an ET such as cuff puncture, tube transection by the needle, accidental extubation, and bronchoscope lesions. The LMA results as an effective and successful ventilatory device during PDT. This may be especially relevant in cases of difficult patient anatomy where improved structural visualization optimizes operating conditions. The intensivist performing PDT should be scrupulous when deciding which method to use. In our ICU the Blue Dolphin PDT with LMA has become the procedure of choice.
